# The gendered politics of the ‘local turn’ in peacebuilding: Acholi traditional justice and gender in post‐conflict Uganda

**DOI:** 10.1111/disa.12677

**Published:** 2025-02-12

**Authors:** Richard Fosu, Eleanor Gordon

**Affiliations:** ^1^ Department of Politics and International Relations Monash University Australia

**Keywords:** gender, local turn, *mato oput*, traditional transitional justice, Uganda

## Abstract

Typically, advancing localised approaches to peacebuilding has evaded critical reflection on the power dynamics and harms that can arise. This paper considers the gender dynamics and gendered harms that can manifest themselves when adopting an uncritical approach to localisation in peacebuilding. To do so, it examines the use of *mato oput* (drinking the bitter root) in post‐conflict northern Uganda, as an example of a localised approach to post‐conflict transitional justice, to investigate these dynamics and harms. It draws from interviews and focus‐group discussions that were conducted in northern Uganda between 2020 and 2022 and engages with critical international relations scholarship on the ‘local turn’ in peacebuilding and the gendered nature of conflict and peace. The paper argues that in patriarchal societies, advancing localisation may entail a return to the traditional or customary order that reaffirms and reifies oppressive gender power relations and helps to consolidate gender inequalities, including unequal access to both security and justice.

## 1 INTRODUCTION

This paper considers the gendered harms that can arise when an uncritical approach to localisation in peacebuilding is advanced, in this case, support for customary or traditional justice approaches to post‐conflict transitional justice (TJ). Feminist critiques of customary TJ mechanisms note that they tend to be gender‐blind and reproduce existing power hierarchies (Dunn, 2017; Rigual, 2018). Essentially, they are sites of contestation where a potentially harmful gendered post‐conflict order is constructed (Dunn, 2017; Björkdahl and Selimovic, 2016). This necessitates, therefore, a careful assessment of the gendered logic and impacts of customary TJ mechanisms.

To investigate the gender dynamics and gendered harms of uncritically advancing a traditional approach to TJ, this paper examines the use of *mato oput* (drinking the bitter root) in post‐conflict northern Uganda. *Mato oput* is an indigenous cultural mechanism used (originally) by the Acholi of Uganda to foster inter‐clan reconciliation in cases where purposeful or accidental death has occurred (Baines, 2007; Finnström, 2008). It includes a process of truth‐telling, accountability, negotiation, compensation, and restoration of relationships following interclan homicide (Baines, 2007; Finnström, 2008).

An exploration of the Acholi restorative mechanism of *mato oput* shows the complex, dynamic, and political character of ‘the local’, including the ways in which it is represented as traditional, authentic, and legitimate, and, through so doing, justifies oppression, exclusion, and harm. We do not suggest that *mato oput* exclusively leads to gendered harms; however, the idea of restorative justice needs problematising since ‘restoration’ connotes ‘reinstatement’ or a ‘return’ to the way things were before conflict (Pavlich, 2002, p. 95), an imagined past or pre‐conflict order that may be exclusionary. As is demonstrated below, practices associated with cultural restoration under *mato oput* are gendered, tending to restore pre‐conflict (gendered) hierarchies.

*Mato oput* seeks to repair social relations and, as a post‐conflict TJ mechanism employed to facilitate progress towards peace, it also aims to restore a pre‐conflict order. A restorative rather than a retributive approach to justice may align more with a feminist approach to building peace that privileges repairing relationships above discipline and punishment (Bell and O'Rourke, 2007). Yet, a process that seeks to repair social relations and restore a pre‐conflict order should invite a critical reflection on power—who exercises it, how it is applied, and what impacts it has. It should also invite critical reflection on how power is imbued within visions of order, deliberating, for instance, in whose interests the vision of order serves.

This paper argues that indigenous/local peacebuilding mechanisms cannot be envisaged outside of local power politics. The local, including everyday/mundane practices that are associated with custom, tradition, and culture, is not apolitical or pre‐political but is shaped by and shapes local (gender) power relations. Thus, localised approaches to peacebuilding, whereby external actors seek to utilise them, are not devoid of the political drivers and interests that often characterise top‐down or externally‐driven interventions. In patriarchal societies, a return to the traditional or customary order may reaffirm and reify oppressive gender power relations and help consolidate gender inequalities, such as unequal access to security and justice. The political interests of powerful groups do not disappear when drawing closer to the community, and nor does the influence of dominant cultural norms and values. Articulation of *mato oput* as a preferred TJ mechanism simultaneously served the political interests of the Government of Uganda (GoU) (seeking to displace accountability and blame), Acholi leaders (seeking to restore legitimacy), and the international community (seeking to bridge the gap between the principle and practice of local ownership).

This paper engages with critical international relations scholarship on the ‘local turn’ in peacebuilding (Richmond and Franks, 2007; Mac Ginty and Richmond, 2013) and the gendered nature of conflict and peace (Cockburn, 2010; Cohn, 2013). It contributes to these substantial corpuses of literature by arguing for recognition of the political character of ‘the local’ and the way in which local approaches to peacebuilding can provide avenues for the reaffirmation and legitimation of gender power relations and gendered inequalities.

The paper articulates and employs a theoretical framework bringing together the concepts of local peacebuilding and feminist peace, along with the theoretical insights of critical peace scholarship. The combination of these understandings allows us to examine the synergies and tensions between the locally‐owned approaches to peacebuilding and gender‐responsiveness and analyse how peacebuilding—and specifically TJ—is informed by and informs power relations in post‐conflict environments. In so doing, it contributes to a body of work that critically engages with the complexities of indigenous and local approaches to peacebuilding (see, for example, Martin, 2021a, 2021b).

## 2 METHODOLOGY

This paper draws from primary data gathered remotely and in‐person in northern Uganda through semi‐structured interviews (n = 40) and focus‐group discussions (FGDs; n = 3) between October 2020 and September 2022. Fieldwork within Uganda was problematised owing to COVID‐19 (coronavirus disease 2019) restrictions, necessitating a remapping of planned activities and the engagement and training of a male and a female in‐country peer researcher.^1^ The peer researchers were tasked with arranging and conducting in‐person interviews and the FGDs. In‐person interviews were supplemented by online interviews carried out by the lead researcher. The lead researcher held two of the semi‐structured interviews via Zoom while the in‐country peer researchers conducted 38 interviews and the three FGDs in‐person.^2^

The data collection approach required deeper consideration of the complexity of security and other ethical implications of doing research in conflict‐affected environments during a pandemic, to ensure that no harm came to the research participants, peer researchers, and others while planning and managing the project remotely. While in‐person fieldwork is generally preferred in qualitative methods in post‐conflict settings that employ interviews, FGDs, and participant observations, the pandemic has demonstrated that ‘neither proximity not [sic] distance are in themselves liberating vectors’ (Mwambari, Purdeková, and Bisoka, 2022, p. 970). Planning and managing fieldwork remotely meant that the lead researcher was removed from the affective and sensory dimensions of in‐person interactions that enrich qualitative data (Krause, 2021). Distance from the field, however, gave the peer researchers the opportunity to exercise meaningful agency in the research process, leading to a more equitable research relationship (Dunia et al., 2023; Fosu, 2024; Mwambari, Purdeková, and Bisoka, 2024).

While remote interviews curtailed access to certain communities and reduced some risks (associated with being seen with a male unknown to the community) while raising others (the researcher being less able to identify or respond to signs of interviewee discomfort, perhaps), the engagement of two in‐country peer researchers facilitated access to members of the community who may have otherwise been unreachable by the (male) lead researcher. The potential risks and limitations of conducting and managing remote interviewees by the lead researcher were mitigated by spending time building a rapport and trust between the lead researcher and the peer researchers, the peer researchers and the interviewees, and subsequently the interviewees and the lead researcher, and by adhering to strict research ethics protocols (Fosu, 2024). The lead researcher had no previous experience of research in northern Uganda and had a limited social and professional network. Therefore, the building of relationships of trust was critical to planning and managing research remotely. The gendered impacts of methodological choices were also considered.

The field sites of Gulu, Lamogi, Pabbo, and Parabongo were chosen because these were places where safe social distancing could be maintained. Interviews were held with leaders of local civil society organisations (CSOs), traditional/cultural leaders, local government officials, and members of the local communities in these areas (ensuring representation by people with a different gender and age and people from disability groups). We interviewed 22 males and 18 females. The FGDs were conducted in Lamogi, Pabbo, and Parabongo. Each FGD involved at least 12 people and included research participants from a broad section of the communities, representing several categories (youth, elderly, women, men, people with a disability, and traditional/cultural leaders). They were asked to reflect on their post‐conflict experiences, including local mechanisms and actors that they believe will be effective in the achievement of sustainable peace in the long term. They were not specifically asked to comment on *mato oput* and its gendered dynamics; however, it emerged as the key theme when participants talked about cultural/indigenous peacebuilding. Responses were coded and analysed thematically. Since the field sites included places known to have been targeted by the Lord's Resistance Army (LRA), involving the capture and killing of civilians, questions that directly related to participants' experiences of abduction were avoided to ameliorate the risk of potential re‐traumatisation.

## 3 CONCEPTUAL/THEORETICAL DISCUSSION OF THE LOCAL TURN AND ITS GENDERED BOUNDARIES

### 3.1 The local turn in peacebuilding

The local turn in critical peace scholarship emanated from a critique of neoliberal peacebuilding as exclusionary, externally‐driven, neo‐colonialist initiatives that can hinder rather than help efforts to build meaningful, sustainable peace (Richmond and Franks, 2007; Mac Ginty and Richmond, 2013). It gained prominence and popularity as an explanation of recurrent peacebuilding failures (Hunt, 2023). This body of work, and the policy it mirrors (UN, 2004), largely argues that peacebuilding endeavours have been limited in their success as they have marginalised or excluded local actors from active engagement in peacebuilding decision‐making. This results in structures, policies, and practices that have little resonance with the needs, experiences, and expectations of local actors. Consequently, they garner little public trust and confidence, are challenged or ignored, and, therefore, lack utility, legitimacy, and effectiveness. For instance, if newly‐established post‐conflict security and justice structures are not aligned with local norms, customs, traditions, and practices, they are unlikely to be widely trusted or employed, and less likely to be able to respond to local security, justice, and peacebuilding needs. Marginalising local actors is also likely to increase their ‘resentment, resistance and inertia’ (Nathan, 2007, p. 3), which can threaten a fragile peace or consolidate an unconstructive reliance on external assistance (Narten, 2009; Gordon, 2014).

### 3.2 Critical engagement with the local

Adherence to the local turn in peacebuilding has, perhaps inadvertently, contributed to the sustenance of a false binary between the local and the international (Paffenholz, 2015; Rigual, 2018). The local–international binary thrives on theoretical and methodological foundations that results in ‘defensiveness’ of the local in relation to the international (Heathershaw, 2013, p. 277). This binary simplifies the local as a homogenous and benevolent whole, leading to its romanticisation (Richmond, 2011a; Mac Ginty, 2015; Watkins and Hasan, 2022). Romanticisation of the local elides the need for a deeper and more nuanced understanding of context, agency, and local politics. There is also a need to recognise the amorphous, dynamic, and heterogeneous character of ‘the local’, acknowledging that is not a distinct, unchanging entity but is subjective, complex, and highly political in character. It can exclude as much as it might include (Kappler, 2015; Randazzo and Torrent, 2021) and may not be committed to peace (Donais, 2018).

Considerable scholarship has been produced explaining who and what constitutes ‘the local’ when referring to locally‐owned approaches to peacebuilding (Nyamnjoh, 2018; Donais, 2021; Philipsen, 2022); however, there is a need for further examination of the complex, dynamic, political character of the way in which local actors interact, exert, and contest power, and define peace. This is because these change over time and across place and are embedded in historical patterns and structures of exclusion and legitimation (Popplewell, 2019). Studies show that local elites can act as ‘governing agents to discipline and regulate other local actors’ to ‘entrench inequalities’ (Obradovic‐Wochnik, 2020, p. 117; see also Millar, 2017; Simangan, 2017). Critically engaging with his own previous research in Uganda, Paul Schulz (2023, pp. 344–346), who has written extensively in favour of locally‐owned approaches to peacebuilding, recently noted that in the light of new evidence, he may have offered an ‘over‐romanticised and idealistic misrepresentation/assessment’ of local/indigenous mechanisms. This inclination to romanticise and simplify the local led to Adam Kochanski (2020, p. 44) urging ‘scholars to curb their enthusiasm until more is known about how these [local] mechanisms work and to what effect’.

This paper argues that it is important to problematise notions of the local and focus on the complexity and political character of local peacebuilding, if the emancipatory potential of critical peace scholarship and the realisation of meaningful, sustainable peace are to be achieved. This means examining power relations within and between a diversity of local actors, and the way in which political power is exercised to extend authority, enhance legitimacy, and disempower marginalised groups under the guise of localisation (Paffenholz, 2015; Kochanski, 2020). As discussed below, the promotion of Acholi traditional justice as a TJ mechanism ostensibly advanced a localisation agenda but served political interests to bolster the legitimacy of Acholi leaders (Branch, 2014; Komujuni and Büscher, 2020) and protect the GoU's sphere of influence and power—by instituting TJ mechanisms that avoided accountability for GoU atrocities against Acholis (Macdonald, 2019). In essence, it is contended that a localised approach to TJ in Uganda presented political opportunities for local actors to make legitimacy claims within an environment emerging from armed conflict and characterised by the renegotiation of and competition over power (Macdonald, 2017; Omach, 2020).

### 3.3 The local through a gender lens

Feminist international relations scholars have established that violence and conflict are gendered, including women, men, girls, boys, and people of diverse gender identities having different experiences during armed conflict (Cockburn, 2010; Cohn, 2013). It follows that different genders have different needs in the aftermath of conflict and that these must find expression in peacebuilding programmes, policies, and processes if the dividends of peace are to be enjoyed equitably (Gordon and Lee‐Koo, 2021). A gender‐responsive approach to peacebuilding can also help to avoid consolidating gender inequalities that, in turn, can undermine prospects for a meaningful and sustainable peace, owing to the links between armed conflict and patriarchal power relations, gender inequalities, and violence against women and girls (Caprioli, 2005; Cockburn, 2010; Cohn, 2013). Björkdahl and Selimovic (2014, p. 202) have argued that a gender‐responsive approach to TJ, a critical part of most peacebuilding endeavours, opens up the space to understand TJ as a ‘site for long‐term construction of the gendered post‐conflict order’.

A locally‐owned approach to TJ in theory aligns with gender‐responsive TJ, given that localisation advances recognition of and engagement with context‐specific needs, norms, and values that may be distinct from those articulated and adhered to by external actors. Integrating traditional or customary justice mechanisms into TJ approaches may seem to provide more opportunities for women to engage with and influence justice outcomes, since women are often perceived to participate more in community‐level structures than in state‐level structures and processes. Yet, as O'Rourke (2008) reminds us, the processes of defining community membership and values can be exclusionary and highly political. While women may participate more in community‐level structures, they may be just as likely to be marginalised from leadership roles in them as they are from state‐level structures (O'Rourke, 2008), and they may suffer the effects of gender bias that permeate both formal and informal structures in patriarchal societies (Allen and Macdonald, 2013). Power relations, including gender power relations, tend to be replicated throughout society, meaning that community‐level structures can be just as exclusionary and disciplinary in nature as state‐level structures (Gordon, Welch, and Roos, 2015)—if not more so in the absence of policies and structures that may protect women's rights and promote gender equality.

Moreover, in practice, there can be a paradoxical tension between the principles of local ownership and gender equality, which underpin a gender‐responsive approach. In patriarchal societies, locally‐owned approaches tend to be shaped by dominant (male) voices and values. Where external actors are involved, there can be a reluctance to advance gender‐responsiveness in places where it is assumed that the principle of gender equality is not embraced (at least by male‐dominated groups). This is often justified by recourse to the argument that it would undermine the principle of local ownership, which is broadly recognised as critical to peacebuilding effectiveness (Gordon, Welch, and Roos, 2015). Even though the principle of local ownership is rarely fully pursued in actual fact, it is believed to determine the extent to which peacebuilding programmes are responsive to (dominant) local needs, accepted by (influential) local stakeholders, and, ultimately, successful (Gordon, Welch, and Roos, 2015). In such contexts, it is unsurprising if local approaches to TJ and peacebuilding more broadly, reflect, protect, and reaffirm gender power relations. The resultant paradox should also be unsurprising: that those who have limited power are frequently, as a result, more vulnerable to harms and less able to access justice or protection. Consequently, advancing a local approach to TJ, without a gender or intersectional lens, can protect and extend the power of dominant groups and further disempower the more marginalised, disguising the power relations at play in post‐conflict societies and further exposing the most vulnerable to insecurity.

Acknowledgement of the multiplicities of ‘locals’ in post‐conflict environments and the power relations between them can help to reveal the ways in which these power relations can simultaneously advance (societal) peace and sustain (gendered) violence. Overlooking the political character of ‘the local’ and the way in which local actors utilise political opportunities to protect and extend their power, helps mask the violence, exclusion, and inequalities that can arise from—and be justified by recourse to—localisation. A gendered analysis of localisation can help reveal these exclusionary processes and their impacts (Hudson, 2009). In the context of TJ, a gendered analysis of traditional justice mechanisms used to help society come to terms with war‐time atrocities—in relation to this paper, the use of *mato oput* in post‐war northern Uganda—can help expose processes that determine whose justice and what order matters.

## 4 LOCALISATION, PEACEBUILDING, AND TRADITIONAL JUSTICE IN UGANDA

### 4.1 Uganda as a locally‐owned peacebuilding success story

Uganda is broadly seen as a story of successful locally‐owned peacebuilding (McDonough, 2008). National ownership of the peace process was facilitated by the fact that the conflict ended with a limited military victory (Titeca and Costeur, 2015). This helped to remove the ambiguity of partners, stakeholders, and ownership that besets many post‐conflict environments where peace has been secured through a truce or negotiations (Donais, 2018). Local ownership of the peace process, beyond elite stakeholders at the national level, was also advanced through indigenous civil society activism. This grassroots action led to the negotiation of the Agreement on Accountability and Reconciliation (AAR) in 2007 during the Juba peace talks. Article 3.1 of the AAR provides that ‘[t]raditional justice mechanisms, such as … Mato Oput … as practiced in the communities affected by the conflict, shall be promoted … as a central part of the framework for accountability and reconciliation’.^3^ Although the Juba peace talks failed, owing to the LRA's refusal to sign the final peace agreement, the GoU has been committed to implementing the terms of the AAR. Traditional mechanisms, such as *mato oput*, mentioned in the AAR, as shown, have received institutional support from both the GoU and the international community (Macdonald, 2019). Consequently, there has been widespread use of Acholi traditional justice mechanisms to help facilitate inclusive and sustainable peace.

### 4.2 Acholi traditional justice and post‐conflict transitional justice

The AAR was the result of Acholi leaders and local CSOs seeking to end the war. They advanced a local and indigenous approach to peacebuilding, including the use of *mato oput*. This Acholi traditional justice mechanism, which, as noted, favours restorative over retributive justice, has been widely utilised in post‐war Uganda as a peacebuilding tool and TJ mechanism.

In policy and practice, TJ is viewed as ‘the full range of processes and mechanisms associated with a society's attempts to come to terms with a legacy of large‐scale past abuses, in order to ensure accountability, serve justice and achieve reconciliation’ (UN, 2004, p. 4). TJ mechanisms typically include prosecutions, truth and reconciliation procedures, memorialisation, reparations, and institutional reform. Prosecutions have tended to consume the most resources and attract the most attention in peacebuilding contexts, reaffirming a retributive rather than a restorative approach to justice (Clark, 2008). Traditional justice in African societies, meanwhile, is embedded in a restorative approach aimed at social repair more than the determination of individual guilt (Tutu, 1999).

Traditional justice can be seen to align with a localised approach to advancing justice and building peace. While traditional justice mechanisms are not widely integrated into externally‐driven approaches to TJ in the aftermath of conflict, they are now broadly recognised by the international community as important mechanisms that provide access to justice by many people who may be removed from formal or state‐located sources of justice (Ansorg and Gordon, 2019; UN, 2004). Indeed, in many countries, the majority of people access security and justice through customary or traditional mechanisms (Baker, 2010; Albrecht and Kyed, 2011). This is often the case where state security and justice sector institutions lack capacity, legitimacy, or reach (beyond urban centres). This can be commonplace in conflict‐affected environments where there are other prohibitive factors, such as cost (including travelling to police stations or courts), time (where the process of accessing justice can be lengthy, compounded by large backlogs of cases), language and literacy (where access requires fluency in another language or literacy skills), and perceived bias (Baker and Scheye, 2007; Baker, 2010; Ansorg and Gordon, 2019; Gordon, 2021).

Formal TJ mechanisms, especially prosecutions in international or hybrid courts, have been criticised for being removed from the people directly affected by conflict, and so doing little to facilitate reconciliation or build domestic institutional capacity to address harms and provide justice. As a result, they can also fuel ‘perceptions of these mechanisms as features of unhelpful, often exploitative, international agendas’ (O'Rourke, 2008, p. 271). In contrast, traditional or customary justice processes are frequently regarded as more context‐specific, less ‘one‐size‐fits‐all’, and more responsive to the specific needs of the local community (O'Rourke, 2008). In recognition of the benefits of traditional justice for TJ, United Nations Secretary‐General Kofi Annan called for the inclusion of local solutions to address war‐time atrocities (UN, 2004).

This increasing recognition within the international community of the value of traditional justice in peacebuilding is also reflected in scholarship, notably work that advances the traditional or ‘cultural turn’ (Bräuchler, 2015), which favours ‘ethnojustice’ (Branch, 2014) or ‘traditional justice’ (Huyse and Salter, 2008) as a means of seeking accountability for past human rights abuses during post‐conflict transitions. Renewed confidence in traditional institutions and indigenous knowledge in peacebuilding has, however, tended to promote uncritically the local as legitimate, unproblematic, and apolitical (Gone, 2017; Kochanski, 2020). A turn towards the traditional, the cultural, or the local must not be simply regarded as in contradistinction to formal, externally‐driven, top‐down interventions. There remains a need to regard the complexity, the embeddedness, and the political character of the traditional, the cultural, and the local. This necessitates recognition that there can be multiplicities of peace, justice, and security, and that dominant visions being advanced can be exclusionary and renegotiate or reaffirm power relations and inequalities.

In Uganda, the Acholi have intensely contested Western notions of justice and peacebuilding as contradictory to their own traditions. Allen (2007, p. 148) argues that while similar claims about international criminal justice have been made in places like Rwanda and Sierra Leone, in Uganda, the ‘lobbying for recognition of local, traditional and restorative judicial approaches has been intense’. As noted, the AAR officially recognises and promotes the localisation of justice and peacebuilding and represents a consensus between the GoU and LRA negotiators on the need to localise justice and peacebuilding; however, it was a consensus of convenience. Macdonald (2017, p. 628) asserts that both parties came to see the International Criminal Court (ICC) intervention as needing to be ‘contained and controlled’, hence, the ARR was a ‘politically‐expedient’ means to avoid war crimes accountability. This was in response to the domestic political dynamics whereby national and subnational political actors (read as traditional authority) contest and resist each other to maintain domestic spheres of influence (Goodfellow and Lindemann, 2013).

Beyond this effort to avoid accountability and control the ICC, at least four other reasons underpin the promotion of the localisation of justice and peacebuilding in Uganda. First, at the national level, the integration of a localised approach to justice and peacebuilding into the ARR arose as a response to the Ugandan state's efforts to avoid local demands on national politics by privileging the localisation of justice and peacebuilding (Quinn, 2012). Second, it was part of a state strategy to place responsibility (and therefore accountability, even blame) with the Acholis. Scholars have shown that the GoU has always framed the conflict broadly as a northern Uganda problem and specifically as a manifestation of the Acholi's innate inclination for violence (Finnström, 2008, 2010; Laruni, 2014). Thus, in response to domestic political dynamics, the AAR promoted Acholi solutions for Acholi problems and government solutions for government problems. It was an effective way for the GoU to avoid accountability for its own atrocities perpetrated during the conflict. This typifies ‘mafia‐type truces’, where a particular agreement aims to entrench existing power structures (Kaldor, 2016, p. 147). Third, from the Acholis' perspective, advancing localised approaches to TJ helped repair the legitimacy of Acholi leaders, damaged after the conflict (Komujuni and Büscher, 2020). Fourth, Acholi traditional leaders saw the ARR as a political opportunity to resist/contest the (Western) international notions of human rights, justice, and peacebuilding, which were seen as contradicting Acholi traditional notions (Armstrong, 2014; Omach, 2020).

This process of and the impetus behind localisation in Uganda underscore the importance of investigating the role of power in post‐conflict recovery and peacebuilding. A politicisation of the local and localisation moves away from romanticisation and towards a more complex, nuanced, and dynamic understanding of context and competing definitions of peace, security, and justice in the scramble for power.

### 4.3 Mato oput and the disciplinary processes of restoration, belonging, and social harmony

*Mato oput* as a TJ mechanism focuses on repairing social relations, after the harms inflicted by war. Okanya, an elderly male research participant, explained the process and the need for social repair as follows:

Basically, it [resolving disputes] is done by having meetings and in that meeting, it is always very conciliatory … because the elders of our culture want people to live together in harmony … in our traditions we share. For instance, if I have a borehole or a tub, it is not for my family members alone; we depend on the resources, which are communally owned; we share wealth, whereby my children will go and fetch water from another person's place; we also have cultural land. Elders consider it important for parties involved in disputes to reconcile when there is grievance. Reconciliation is done by meeting aggrieved parties together and finding out what went wrong … in our tradition, there are number of mechanisms used to identify some of the problems that could cause grievances.^4^

Otiko, an indigenous CSO leader, reiterated the communal foundation and the need for social repair:

Peacebuilding requires reconciliation. In Africa, we live with one another. You are not strong without somebody else. So, we need to rebuild the lost relationships, because during the war people fought one another. You were made to kill members of your own family. You are assigned to another community to kill. So, it is about facilitating mediation dialogue.^5^

Part of this repair involves seeking to return to an imagined past before these harms and fissures occurred: a past that exists in the imagination but still forms the bedrock of many of the cultures, norms, and practices that persist among Acholi people (Quinn, 2010). Otiko remarked: ‘if conflicts existed before and they were resolved, why don't we resolve it the same way?’.^6^ He continued:

In the natural context, conflicts started way back, and they [Acholis] were dealing with it. What structures were they using to deal with these conflicts? We need to reinstate the structures. It is all about reinstating the structures and building their capacity to help people understand and repair their communities.^7^

In this ‘natural context’, peacebuilding is a process of ‘reconciliation’, ‘compensation’, and ‘forgiveness’. Participants explained this conception of peacebuilding as the ‘Acholi way of life’, ‘our tradition’, and ‘our culture’. According to Otim:

Peacebuilding is a process of reconciliation, because maybe if you have killed my beloved one and we want to build peace with you or with your family members, first we should begin with reconciliation. When we talk about reconciliation it includes telling the truth and accepting that you have done wrong and asking for forgiveness. Secondly, after reconciling, then we could now see how we can start developing. If in the process of war, I have taken your car or taken all your land, then as part of the peacebuilding, I will say now that we have reconciled and let me return to you the wealth that I've taken from you or let me compensate you so that you're able to start from where I was before the incident happened. This is what I count as peacebuilding.^8^

This envisaged past and the reaffirmation of the social order that predated disruption and threat is a political order and not a natural order, as may be presented. It tends to serve the interests of powerful actors within a community (Sseremba, 2023). This pre‐conflict social order can be oppressive and exclusionary and may be resisted by those who may have accessed new opportunities during the conflict (Branch, 2014). It is also an order which may have contributed to the outbreak of conflict in the first place (Allen and Macdonald, 2013). This calls for problematisation of cultural restoration given that tradition changes over time and there is not a singular, agreed fixed vision of the past.

When an offence is committed in Acholiland, the first option is ‘family‐to‐family’ negotiation, as Margaret, an elderly female research participant, pointed out.^9^ Thus, ‘no good Acholi pursues justice alone’ (Porter, 2018, p. 179). Many of our research participants argued that any form of justice that does not prioritise communal relationships or ‘social harmony’ threatens peace (Porter, 2012). Santo, a male community leader, underscored that weakened communal justice mechanisms after conflict are a threat to peace:

Under the laws in the community now, most times when young children do something wrong, they are taken straight to court and imprisoned and yet it could be solved at family level. Even if you try to negotiate with the family, you are also arrested … there are so many young people in prison because they have eliminated family‐to‐family negotiation. Those days you negotiate with your colleagues and apologise. But now, children are taken straight to court, which is stressing our children. If you go to prison and ask how old these children are, you will find that they are very young people. They sin once and are imprisoned. That is something not going well with the peace that we have.^10^

Patrick and Oloya, community and cultural leaders respectively, lamented the state's intrusion into communal justice and saw it as a threat to the reciprocal nature of communal justice in Acholi. According to Oloya, long‐term peace is contingent on the government relinquishing authority over justice to ‘elders at home to try solving certain issues’.^11^ She said that ‘people go straight to the government’ to seek redress for offences, rather than ‘giving authority for such things to be handled at home’.^12^ For Patrick, ‘community leaders’ should be allowed initially to ‘handle matters at [the] family level even if someone has died’.^13^ In his words:

In the past, we could first sit and talk, and if someone has done something intentionally, then they acknowledge it, but if they have not done it intentionally, then it is not even taken to court. That is what we used to do so I feel they should reverse to it because if you arrest someone's child, the hate is so intense as if you have just killed them. The moment you imprison them, then they would do the same to you the moment your child does the same, they would remind you how you did not forgive their own.^14^

This conception of justice as restorative in Acholiland often becomes disciplinary because a *good* Acholi does not take matters from ‘home people’ and give them to the ‘people of human rights’ since the latter lack ‘moral jurisdiction’ (Porter, 2012, pp. 84–96). Moral jurisdiction lies with ‘in‐laws, clan leaders or other respected community members’ (Porter, 2012, p. 87). As a means of repairing social relations, *mato oput* places communal justice above individual justice. While this may help facilitate progress towards social harmony and peace in the community, without attendance to power relations, it can also inadvertently privilege the needs of powerful actors within the community ahead of those of marginalised or disenfranchised actors. Communal justice, in other words, tends to benefit those in positions of power within the community, and can reaffirm power relations and inequalities. As Allen and Macdonald (2013, p. 15) have argued, ‘customary justice can elevate the goal of community harmony above individual rights and freedoms in worrying ways’. The restoration of order and community harmony, therefore, may be order and harmony for some at the expense of security and justice for others.

Typically, *mato oput* is used in disputes between individuals/families so there are concerns about whether traditional justice can deal with mass war crimes (Liu Institute for Global Issues, Gulu District NGO Forum, and Ker Kwaro Acholi, 2005). *Mato oput* is traditionally only applicable to murder cases, not other serious crimes such as large‐scale human rights violations, including the systematic killing, abduction, and rape that occurred during the conflict in northern Uganda (Baines, 2007; Ensor, 2013). Ensor (2013, p. 185) notes that ‘[t]here is also concern that Acholi traditional justice practices may be unsuitable for addressing crimes committed outside the Acholi region, or against non‐Acholi victims and their families not subject to local traditional authority’.

Additionally, while Acholi cultural narrative framed former abducted LRA fighters as ‘complex political perpetrators’ (Baines, 2009) and thus ‘complex victims’ deserving of forgiveness (Omach, 2016; Baines, 2017), community sentiment regarding forgiveness and reintegration has been different (Kiconco and Nthakomwa, 2018; Kiconco, 2022). As Anderson (2009, p. 72) argues, ‘the former preconditions for *mato oput* have been largely dismantled with the humanitarian crisis that ensued in northern Uganda’ (see also Dolan, 2009). Moreover, in the post‐conflict settings, the victim's family may not know the perpetrator, and clan unity may not be as strong as it once was (Anderson, 2009). And there is also pervasive deprivation, which has compromised the ability of perpetrators to provide reparations. Furthermore, in Uganda, ‘conditions have devolved so much that Acholi youths are disconnected from their cultural roots, and local justice measures might not hold meaning for them like they did for their parents’ generation’ (Anderson, 2009, p. 72; see also Ensor, 2013).

Another concern is that the war undermined the legitimacy of cultural institutions and leaders, resulting in the ‘invention of traditional justice’ (Allen, 2007), the perverse interpretation of culture, and corruption (Baines, 2007; Quinn, 2022). Okwir, an indigenous CSO leader, pointed out that:

The war disempowered these institutions such that even knowledgeable people in these institutions are lacking. Some of the current chiefs that we have do not know the processes and practices when it comes to healing and reconciliation rituals. So, the question of capacity is a problem.^15^

He further noted that wartime displacement and despair impoverished cultural leaders, leading to questionable practices in the name of culture:

Many chiefs and cultural leaders have become poor because of the war. Those days they were wealthy, but the war affected everyone … because they are impoverished, they started bending cultural norms … to ask for payment, which people think is weird. It has become so easy for them to be manipulated.^16^

As a means of reasserting lost legitimacy, Acholi cultural leaders were able to use *mato oput* to reaffirm and consolidate their power as well as rearticulate or reform the boundaries of community membership and exclusion. Tradition, culture, norms, and values were reformed and rearticulated through the practice of *mato oput*, serving to reconstitute the Acholi way of life, reaffirm the legitimacy and power of cultural leaders, and exclude—and justify the exclusion of—marginalised groups and individuals. The promotion of a localised approach to TJ, through the incorporation of *mato oput*, thereby provided a political opportunity for cultural leaders to reconstitute their legitimacy and, in so doing, advance a politics of belonging (Stewart, 2017, 2021).

Restoration has become disciplinary—a means of simultaneously repairing social relations but in a way that might be oppressive, even violent. The disciplinary process of seeking compliance by community members through the discourses and practices of the ‘Acholi way of life’ and determining who belongs and who does not may at first appear to be at odds with a traditional justice process that privileges repair and restoration above retribution and punishment. However, the (re)making of order is always political. This power is both enabling and oppressive, benefitting those who can make legitimate claims to tradition, culture, and custom, and harming those who may be on the margins of that community.

As we discuss below, this order‐making is gendered. Notwithstanding the value of repairing and preserving social relations, especially in societies recovering from the effects of armed conflict and war‐time atrocities, it must be recognised that these benefits can come at the expense of those who may be marginalised. Through a gender analysis of Acholi traditional justice practices, we aim to examine critically the ‘local turn in TJ’ (Kochanski, 2020) and peacebuilding broadly as a ‘site for long‐term construction of the gendered post‐conflict order’ (Björkdahl and Selimovic, 2016, p. 70; see also Björkdahl and Selimovic, 2015). The forward‐looking approach to social repair, focused on building peaceful relationships, can yield harmful even violent outcomes for the disenfranchised. Importantly, a gender analysis of Acholi traditional justice mechanisms can help reveal the power dynamics that are often obscured in localised approaches to post‐conflict justice. It can also shed light on the power relations and inequalities that produce and are reproduced by these mechanisms. Thus, it can promote more critical engagement with the principle of localisation in peacebuilding. A gender analysis of local norms and practices therefore offers a space in which to observe oppressive local structures and provide analytical tools to de‐romanticise the local and help realise an emancipatory peace.

## 5 *MATO OPUT* AND GENDER

Women suffered deeply during the conflict in northern Uganda, often violently targeted by state forces and the LRA when they were fulfilling caring and household responsibilities, such as collecting water and firewood (Quinn, 2010). Women and girls were forcibly recruited as ‘wives’ or sex slaves of LRA fighters and suffered other forms of conflict‐related sexual violence (CRSV), including rape/gang rape, sexual slavery, and reproductive violence (Nabukeera‐Musoke, 2009). As an indication of the prevalence of CRSV, the United Nations Children's Fund (UNICEF, 2005) reported that more than 60 per cent of women and girls in the largest internally‐displaced persons camp (Pabbo in Gulu District) had experienced some form of sexual or gender‐based violence (SGBV). A high prevalence of SGBV against women and girls, including child and forced marriage, also persists in post‐conflict Uganda (Bukuluki et al., 2017). Given the significant war‐time harms suffered by women and girls—and the persistent threat of SGBV—there is a particular need for TJ mechanisms to address them.

Despite the promise of *mato oput* as a localised approach to TJ, it has been criticised as being gender‐insensitive (Anderson, 2009), excluding or only assigning a minor role to female participants (Ensor, 2013), and not dealing with the root causes of crimes committed against women and girls (Mazurana and Carlson, 2010). There is also a requirement for a more prominent role for women in such TJ processes, especially if these processes are to be responsive to their specific needs (Ogora and Murithi, 2011). Male dominance of *mato oput* can also result, as with other traditional justice mechanisms, in male leaders using such processes to further their legitimacy, control, and/or interests. For instance, male elders have used *mato oput* to discipline returning former LRA combatants, particularly youths (Allen and Macdonald, 2013; Branch, 2014). In northern Uganda, and elsewhere, local/indigenous TJ processes have, in effect, been employed to restore the power and legitimacy of male elders, damaged or fractured due to the armed conflict. The return to the pre‐conflict order that *mato oput* seeks is, therefore, a return to a patriarchal order and an order that serves the political interests of (male) elders. It can thus help to perpetuate gender inequalities and exclude those (women and youths) who may challenge cultural restorative mechanisms (Hodgson, 1996; Opinia and Bubenzer, 2011; Branch, 2014).

Women (and youths) strategically engage with, support, contest, redefine, and sometimes reject customary norms and institutions (Hodgson, 1996; see also Dunn, 2017).^17^ Yet, as Bell and O'Rourke (2007, p. 41) have argued, ‘the notion of “restoring” that lies at the heart of [the restorative] conception of justice speaks of a return to a set of relationships that for women may have been fundamentally unjust’. Gender justice in this order may be seen as contradicting Acholi cultural restorative practices. In an interview, Okwir, an indigenous CSO leader, described the concept of gender as a challenge to Acholi cultural restoration:

It looks like the concept of gender is conflicting with our traditional way of doing things. Cultural leaders, chiefdoms, and local institutions are the custodians of the traditional ways … and since gender is a new concept, they don't know how to go about it. This is what I can explain to you concerning what is challenging the traditional institutions.^18^

Similarly, Okot, a male employee of an indigenous CSO, explained that his organisation's peacebuilding activities are informed by gender justice norms because peacebuilding ‘is all about paying attention to the unique gender differences during the conflict.^19^ Yet, he emphasised that ‘the Western world value is not exactly … the African value’,^20^ adding:

We are using donor money mostly from the Western world. It has gotten strings attached. It has its ways, what they value, so sometimes you are forced to do that [promote gender equality] because they [Western donors] are giving you the money. So, we tend to implement programmes that are not cultural to our community, and this has also affected our cultural institutions in governing their people.^21^

The comments of Okwir and Okot can be illuminated by situating them within (feminist) scholarship on Uganda, especially that which examines traditional responses to gender‐based violence, including rape (Anderson, 2009; Porter, 2012, 2015a, 2015b, 2019). Porter (2015a, p. 82) contends that ‘the primary moral imperative in the wake of wrongdoing is not punishment of the perpetrator or individual victim's rights but the restoration of social harmony’. Porter (2012) found in her studies that all victims of marital rape thought their husbands should be punished; however, they neither reported them to security providers nor sought justice. Rape victims, who felt that their perpetrators deserved punishment, wanted a third party, mainly a trusted male relative, to talk to them, which was seen as adequate punishment. Other victims who were raped by a relative saw traditional cleansing and elders' admonishment as appropriate punishment or a just response. Of course, a disinclination to report rape remains prevalent in most, if not all, societies globally, although Porter (2012) regards a reluctance to seek punishment in Uganda as being the result of enormous pressure to maintain social harmony. It is through the processes of maintaining social harmony that Acholi cultural leaders retained their political and regulative powers. Thus, the pre‐conflict Acholi order was anchored to—and benefitted—male Acholi elders and other lineage‐based authorities (Branch, 2014; Laruni, 2014).

In such patriarchal societies, women may be disadvantaged by mechanisms that seek to restore communal harmony, and they may be less likely to influence the development of these mechanisms in a way that responds to their specific security and justice needs. The shaping of norms and values is most heavily influenced by dominant members of a community, who are also more likely to be able to characterise such norms and values as traditional and for the good of the community. Indeed, some women interviewed for this project noted that the breakdown in Acholi cultural norms/authority has negatively affected the socialisation of children and communal harmony, necessitating re‐acculturation. This process of norm articulation and reaffirmation as a result also helps reaffirm the legitimate authority of these dominant members, while further marginalising—and justifying the marginalisation of—those who may not fit or may, directly or indirectly, challenge the logic or fairness of the practices and processes to which these norms and values give rise. Branch (2014, p. 622) notes that while older ‘men have seen their authority and status within Acholi society wane, women, and to a lesser extent youth, have seen their economic, social and political status rise’. Women have also benefitted from the economic and educational opportunities brought by humanitarian interventions; however, these opportunities have also become sources of challenges, even violence, in the context of efforts to reaffirm traditional gender norms and practices and reimpose an idealised pre‐conflict order.

Many female participants highlighted during interviews that while they have gained skills from various peacebuilding initiatives, their financial independence has become a threat to their homes and marriages. Abonyo, a female participant, recounted a story concerning her husband's disapproval of her involvement in a skills development programme by a local CSO. He told her that ‘if she thinks the peacebuilding activities she is engaging in will marry her, then let her marry peacebuilding activities’.^22^ Her husband got angry and assaulted her. Some indigenous CSO leaders explained that such situations are prevalent and emanate from changing gender roles. Odwar, a female employee of an indigenous CSO, stated:

When the women we support become economically independent, they then begin to get challenges in their marriages. Their husbands want to control their resources that they have acquired through their own hard work. This becomes a cause of conflict in their homes…. In our culture, men are expected to provide, so in situations where the woman controls more resources than her husband, the man suddenly wants to take the resources.^23^

While the conflict in northern Uganda has altered gender roles and practices in Acholi society, there is much resistance to this change. Women's greater economic power and participation in public and political life in the aftermath of war tends to provoke violent resistance by those who may feel emasculated, disempowered, or threatened as a result, in an effort to reassert their manhood and maintain power and control (Sideris, 2002). Hence, a return to an idealised past serves as a form of communal gatekeeping: as a disciplinary process seeking compliance, reminding community members of the norms and practices that govern acceptable behaviour, and determining who belongs and who does not. From a feminist perspective, a return to the pre‐conflict order may reaffirm gender inequalities and undermine women's new‐found opportunities. A return to the pre‐conflict order often, therefore, implies a return to a gendered, patriarchal order (MacKenzie and Foster, 2017). Consequently, a restorative vision of Acholi traditional justice practices should call for critical reflection on the kind of order it seeks because it ‘can end up extending forms of unaccountable, patriarchal power within Acholi society’ (Branch, 2014, p. 608). Therefore, not all cultural norms or practices should be revived (Baines, 2015). The gendered power relations that inform and are reproduced by traditional justice mechanisms sustain inequities of power and unequal access to resources and justice.

It is striking that all of the indigenous CSO actors interviewed—both male and female—provided no indication that such local norms are problematic. Rather, they seemed to support and promote such practices as ‘our culture’ and the ‘traditional way’ of doing things. This is analytically relevant given the conception of the indigenous CSO in the local turn scholarship as possessing the ability to achieve emancipatory peace (Richmond, 2009, 2011a, 2011b). Indigenous CSO actors are seen as norm entrepreneurs (Groß, 2015) or ‘people interested in changing social norms’ (Sunstein, 1996, p. 909). This discourse suggests that indigenous CSOs (should) open up space for civil conversations, including challenging oppressive and discriminatory local norms. Yet, indigenous CSOs can be purveyors and preservers of local norms, including exclusionary and oppressive ones (Popplewell, 2019).

When discussing the issue of gendered violence in Acholiland, Okello, a female participant, noted that many indigenous CSOs—including female‐led ones—‘are giving bad advice to husbands on domestic violence’.^24^ She explained that they ‘only talk about [focus on] women even if a man is wrong … they [the CSOs] do not talk to men about their abusive behaviours’.^25^ Other participants noted that some indigenous CSOs have concentrated on training women in prevention tactics to reduce domestic violence. The conception of domestic violence prevention as a women's responsibility is built on the assumption of the Acholi traditional way, wherein women unquestionably obeyed their husbands (Branch, 2014). Restorative peace mechanisms that accept such unequal gender power relations as traditional risk reversing the gains women can make during conflict (Bell and O'Rourke, 2007). Therefore, placing the burden of domestic violence prevention on women, as suggested by the activities of some indigenous CSOs, is problematic. The promotion of preventative activities as Acholi culture by indigenous CSOs closes the space to challenge oppressive local norms. This could lead to the normalisation of inter‐personal violence, especially in a context where marriage constitutes an important source of women's identity and economic security (Kiconco and Nthakomwa, 2018). The following remarks of Auma, a middle‐aged female community leader involved in ‘helping’ women experiencing domestic violence, illustrate this point:

As a mother, I have been advising my fellow mothers that in case a man disturbs them, I let them know that men are like that … your husband already wasted your time so there is no need leaving with your children to your parents' home. So, it's important that you stay where you are married … personally, I already made up my mind in that line.^26^

## 6 CONCLUSION

This paper has considered the gendered harms that can arise when an uncritical approach to localisation in peacebuilding is advanced, in this case support for customary or traditional justice approaches to post‐conflict TJ. To investigate the gender dynamics and gendered harms of uncritically promoting a traditional approach to TJ, this paper has assessed the use of *mato oput* in post‐conflict northern Uganda. Typically, advancing localised approaches to peacebuilding has evaded critical reflection on the power dynamics and harms that can arise. This paper contributes to existing scholarship on the ‘local turn’ in peacebuilding, specifically TJ, by drawing attention to the political character of the local, which may simultaneously reaffirm and consolidate power relations (including gendered power relations). This can give rise to harms, as well as build local resilience and resist external exploitative or oppressive tendencies that have tended to dominate peacebuilding to date.

As stated, an investigation of the Acholi restorative mechanism of *mato oput* shows the complex, dynamic, and political character of ‘the local’, including the ways in which it represents itself as traditional, authentic, and legitimate, and, through so doing, can justify oppression, exclusion, and harm—as well as facilitate much‐needed restorative processes, particularly in the aftermath of armed conflict. Articulation of *mato oput* as a preferred TJ mechanism concomitantly served the political interests of the GoU (seeking to displace accountability and blame), Acholi leaders (seeking to restore legitimacy), and the international community (seeking to bridge the gap between the principle and practice of local ownership). Indeed, customary mechanisms may be the only means for many conflict‐affected communities to mend broken social ties, rebuild communal relationships, and reconstruct their lives. Yet, the political interests of powerful groups do not disappear when drawing closer to the community, and nor does the influence of dominant cultural norms. The value of indigenous/local peacebuilding mechanisms cannot be envisaged outside of local power politics. In patriarchal societies, a return to the traditional or customary may reaffirm and reify oppressive gender power relations and help consolidate gender inequalities, including unequal access to security and justice. Recognition of the gendered harms that can arise from customary approaches to TJ and broader peacebuilding is not to argue for a resurgence of externally‐driven interventions, but to urge attentiveness to the political character and (gendered) power dynamics that permeate societal structures at all levels, including the local or community levels. Consequently, to help realise the emancipatory potential of critical peace scholarship and build meaningful, sustainable peace, it is necessary to problematise notions of the local and pay regard to the complexity and political character of local peacebuilding.

## ETHICS STATEMENT

This paper reports analysis of primary sources. The ethics of data collection and analysis was approved by the Monash University Human Research Ethics Committee. Persons from whom data were collected gave their free, prior, and informed consent. Their data have been kept confidential and used anonymously.

## FUNDING

No direct funding was received for the drafting of this article. However, the first author was a recipient of Monash University's Faculty of Arts International Postgraduate Research Scholarship Scheme, covering tuition and health insurance. He also acknowledges receipt of a stipend from the Australian Government Research Training Program.

## Data Availability

Research data are not shared.

## Endnotes

^1^
Both peer researchers are Acholi Ugandans with lifelong experience of living and working in Gulu in northern Uganda. They each have at least 10 years' experience as researchers. Their positionality as Acholi Ugandans who have lived and worked in Gulu means that they have a wide and relevant social and professional network, critical to fieldwork success.

^2^
A comprehensive discussion of managing and conducting in‐person and online fieldwork during the COVID‐19 pandemic for this project has been published elsewhere: see Fosu (2024) for a discussion of the methodological choices and positionality of the lead researcher and in‐country peer researchers, as well as the ethical challenges and how they were addressed.

^3^
For more information, see https://ucdpged.uu.se/peaceagreements/fulltext/Uga%2020070629.pdf (last accessed on 22 January 2025).

^4^
A male participant, Gulu city, 16 July 2021.

^5^
Indigenous CSO leader, Gulu city, 26 May 2021.

^6^
Indigenous CSO leader, Gulu city, 26 May 2021.

^7^
Indigenous CSO leader, Gulu city, 26 May 2021.

^8^
A male councillor, Gulu District Local Government, 23 December 2021.

^9^
A female participant, Gulu city, 20 September 2021.

^10^
A male community leader, Gulu city, 22 September 2021.

^11^
A female cultural leader, Gulu city, 6 May 2021.

^12^
A female cultural leader, Gulu city, 6 May 2021.

^13^
A male cultural leader, Gulu city, 17 July 2021.

^14^
A male cultural leader, Gulu city, 17 July 2021.

^15^
Indigenous CSO leader, Gulu city, 8 December 2020.

^16^
Indigenous CSO leader, Gulu city, 8 December 2020.

^17^
We are grateful to the second anonymous peer reviewer for directing us to these authors and their arguments.

^18^
Indigenous CSO leader, Gulu city, 8 December 2020.

^19^
A male employee of an indigenous CSO, Gulu city, 17 December 2020.

^20^
A male employee of an indigenous CSO, Gulu city, 17 December 2020.

^21^
A male employee of an indigenous CSO, Gulu city, 17 December 2020.

^22^
A female participant, Gulu city, 23 December 2021.

^23^
A female employee of an indigenous CSO, Lamogi, 20 October 2021.

^24^
A female participant, Gulu city, 4 June 2021.

^25^
A female participant, Gulu city, 4 June 2021.

^26^
A female community leader, Gulu city, 7 June 2021.
